# In Vivo Evaluation of Diode Laser Use in Lingual Frenectomy: A Histological and Histomorphometric Study

**DOI:** 10.3390/dj14040209

**Published:** 2026-04-03

**Authors:** Claudia Marcia de Moraes Souza, Adriana Terezinha Neves Novellino Alves, Rodrigo Figueiredo de Brito Resende, Juliana Pires Abdelnur, Jose de Albuquerque Calasans-Maia, Carlos Fernando Mourão, Jamil Awad Shibli, Jose Mauro Granjeiro, Monica Diuana Calasans-Maia

**Affiliations:** 1 Post-Graduation Program in Dentistry, Fluminense Federal University (UFF), Niteroi 24220-140, RJ, Brazil; dra.claudiamms@gmail.com (C.M.d.M.S.); juabdelnur@hotmail.com (J.P.A.); 2 Oral Diagnosis Department, Fluminense Federal University (UFF), Niteroi 24220-140, RJ, Brazil; aterezinhanovellino@gmail.com; 3 Oral Surgery Department, Fluminense Federal University (UFF), Niteroi 24220-140, RJ, Brazil; rodrigoodonto21@hotmail.com; 4 Orthodontics Department, Fluminense Federal University (UFF), Niteroi 24220-140, RJ, Brazil; josecalasans@gmail.com; 5 Department of Basic and Clinical Translational Sciences, School of Dentistry, Tufts University, Boston, MA 02111, USA; carlos.mourao@tufts.edu; 6 Dental Research Division, Department of Periodontology and Oral Implantology, University of Guarulhos (UNG), Guarulhos 07023-070, Brazil; 7 Faculdade Israelita de Ciências da Saúde Albert Einstein, Hospital Israelita Albert Einstein, São Paulo 05653-120, Brazil; 8 Department of Oral Medicine, Infection, and Immunity, Division of Periodontology, Harvard School of Dental Medicine, Boston, MA 02115, USA; 9 Research Unit Periodontology and Oral Microbiology (P&OM), Department of Oral Health Sciences, KU Leuven, B-3000 Leuven, Belgium; 10 National Institute of Metrology, Quality and Technology (INMETRO), Duque de Caxias 20261-232, RJ, Brazil; jmgranjeiro@inmetro.gov.br; 11 Clinical Research Laboratory, Dentistry School, Fluminense Federal University, Niteroi 24220-140, RJ, Brazil

**Keywords:** frenectomy, high-power laser, diode laser, rats, histological evaluation

## Abstract

**Background/Objectives**: Morphological alterations of the lingual frenulum may impair sucking, speech articulation, and tongue mobility. In such cases, frenectomy is considered the most effective therapeutic approach. High-power lasers have been increasingly adopted due to their precision and reduced surgical trauma. This study aimed to compare the effects of frenectomy performed with a cold scalpel, electric scalpel, and diode laser in Wistar rats. **Methods**: Forty-five female rats, approximately six months old and weighing 250–300 g, were randomly allocated into three experimental groups (*n* = 15) according to the surgical technique used. Each group was further divided into three subgroups (*n* = 5) based on postoperative evaluation periods of 1, 3, and 7 days. After surgery, animals were euthanized at the predetermined time points, and tissue samples from the operated area were collected for histological analysis. Specimens were processed histologically, and sections were analyzed descriptively and semi-quantitatively for inflammatory response. **Results**: All surgical techniques produced similar inflammatory responses across the evaluated periods, with no statistically significant differences among groups. The inflammatory infiltrate was predominantly acute, characterized by the presence of neutrophils, lymphocytes, and macrophages, with scarce plasma cells and no multinucleated giant cells. Although the cold scalpel group showed greater variability in macrophage response over time, the electric scalpel and diode groups exhibited more consistent inflammatory patterns. **Conclusions**: All evaluated techniques were biologically equivalent with respect to the initial inflammatory response, with the electric scalpel and high-power laser showing slightly greater temporal stability.

## 1. Introduction

The lingual frenulum is a dynamic anatomical structure [1], typically observed as a thin mucosal fold that connects the ventral surface of the tongue to the floor of the oral cavity. It plays a fundamental role in regulating tongue stability and mobility, thereby contributing to essential functions such as swallowing, feeding, and speech [2]. Variations in its morphology—including alterations in shape, length, thickness, site of insertion, or tissue composition—may compromise these functions and result in clinically significant functional impairments.

Such morphological alterations may characterize a condition known as ankyloglossia, commonly referred to as tongue-tie. Ankyloglossia is defined by an abnormal insertion of the lingual frenulum and is frequently associated with the persistence of embryonic tissue that fails to undergo complete apoptosis during development [1]. Anatomically, the frenulum extends from the ventral surface of the tongue to the lingual aspect of the mandibular alveolar ridge, typically in the region of the lower incisors. The reported prevalence of ankyloglossia varies considerably in the literature, ranging from 0.1% to 10.7%, a discrepancy largely attributed to the absence of standardized diagnostic criteria [3,4]. Although its etiology remains incompletely understood, higher prevalence rates have been consistently reported among males and individuals with a positive family history [3].

From a histological perspective, the lingual frenulum is composed of stratified squamous epithelium, which is keratinized in the region of the attached gingiva and non-keratinized in the vestibular portion. The underlying connective tissue contains abundant collagen and elastic fibers, adipose tissue, muscle fibers—particularly from the superior portions of the genioglossus muscle—and blood vessels lined by stratified epithelium [5,6,7,8].

Clinically, lingual frenula may be classified into four subtypes based on their length: Class I (12–16 mm), Class II (8–11 mm), Class III (3–7 mm), and Class IV (<2 mm) [7,8,9]. Alterations may involve not only length but also thickness, insertion site, and tissue composition, with functional consequences that are strongly influenced by the patient’s age group [9].

In newborns and infants, lingual frenulum abnormalities may interfere with effective breastfeeding. In children and adults, ankyloglossia has been associated with impairments in mastication, swallowing, phonation, and articulation. Additionally, it may contribute to periodontal complications, such as gingival recession and difficulties in maintaining oral hygiene; orthodontic alterations, including misalignment of the lower incisors; prosthetic instability in removable dentures; and psychosocial consequences, particularly those related to impaired verbal communication and reduced self-esteem [7,9,10,11,12].

According to Mazzoni, the prevalence of ankyloglossia in the general population ranges from 4.2% to 10.7%, while reported rates in newborns vary from 0.52% to 21%. The condition predominantly affects males, with a male-to-female ratio ranging from 2:1 to 2.6:1. Such variability is primarily attributed to differences in diagnostic criteria and assessment methodologies across studies [13]

The therapeutic approaches most recommended for the correction of lingual frenulum alterations include frenotomy, frenectomy, and frenuloplasty. These surgical procedures involve partial excision, complete excision, or repositioning of the frenulum, respectively, and aim to restore normal function and prevent associated complications [14]. Such interventions are typically performed in outpatient settings under local anesthesia. A variety of surgical techniques have been described in the literature, including those proposed by Archer, modified Archer, Chelotti, Wassmund, Mead, and Howe, as well as techniques employing electrosurgery and high-power laser devices [15].

The selection of the surgical approach should consider several factors, including the patient’s age, the anatomical characteristics of the frenulum, the clinician’s experience, and logistical as well as economic considerations. Technological advances have contributed to making frenectomy an increasingly safe and predictable procedure, with reduced morbidity. Conventional cold scalpel techniques remain widely used due to their low cost and technical simplicity; however, they are associated with increased intraoperative bleeding, greater postoperative discomfort, and the frequent need for suturing [16]. Electrosurgical techniques provide effective hemostasis and reduce operative time but may induce greater thermal injury to adjacent tissues.

More recently, high-power surgical lasers have been introduced as an alternative modality, as they offer advantages such as reduced intraoperative bleeding, decreased anesthetic requirements, elimination of sutures, and improved patient comfort during the postoperative healing period. Nonetheless, their clinical implementation is limited by the high cost of equipment and the requirement for specialized training [17].

Over the past three decades, laser technology has become increasingly integrated into modern dental practice, prompting extensive investigation into the various types of lasers, their clinical applications, efficacy, limitations, and relative advantages and disadvantages—particularly with respect to high-power surgical lasers [18]. Laser-assisted surgery is often described as a minimally invasive and time-efficient technique, with an average operative duration of approximately 2–5 min. It enables enhanced visualization of the surgical field due to effective hemostasis, allows precise tissue incision and coagulation, reduces anesthetic requirements, promotes immediate decontamination of the surgical site, minimizes postoperative edema, and consequently results in reduced postoperative discomfort.

Currently available high-power laser systems include those primarily indicated for soft-tissue procedures, such as neodymium-doped yttrium aluminum garnet (Nd:YAG), carbon dioxide (CO_2_), and diode lasers, as well as lasers suitable for both hard- and soft-tissue applications, including erbium-doped yttrium aluminum garnet (Er:YAG) and erbium-doped yttrium scandium gallium garnet (Er:YSGG) lasers [19,20,21].

In addition to surgical management, the treatment of ankyloglossia frequently requires adjunctive speech-language assessment and myofunctional therapy to reestablish appropriate speech articulation patterns and optimize orofacial functional performance [9].

Despite the increasing use of diode lasers for lingual frenectomy, the biological response of oral tissues to laser incision remains a subject of debate. While laser surgery is often associated with reduced intraoperative bleeding, decreased postoperative discomfort, and a potentially lower inflammatory response, concerns persist regarding the possibility of thermal damage to adjacent tissues. This thermal effect may influence the early inflammatory phase of wound healing, which is a critical determinant of subsequent fibroblast activity, collagen deposition, and ultimately scar formation and fibrosis. Therefore, understanding the early histological inflammatory response is essential to clarify whether laser-based procedures truly promote more favorable healing compared with conventional techniques. Based on this rationale, the present study hypothesized that diode laser frenectomy would produce a distinct early inflammatory and histomorphometric profile compared with conventional scalpel surgery, potentially influencing the tissue remodeling process.

Notwithstanding the widespread clinical use of various surgical modalities for frenectomy, the cellular and tissue-level responses elicited by different techniques remain incompletely characterized. This gap in knowledge highlights the need for well-designed comparative studies to elucidate their biological, inflammatory, and healing-related effects on oral tissues.

## 2. Materials and Methods

### 2.1. Ethical Considerations

This study was reviewed and approved by the Ethics Committee on the Use of Animals of the Federal Fluminense University (CEUA/UFF; approval no. 4157050324; approval date: 10 May 2024). All procedures involving animal breeding, handling, treatment, maintenance, and euthanasia were conducted in strict accordance with the Brazilian Guidelines for the Care and Use of Animals for Scientific and Educational Purposes (DBCA), established by the National Council for the Control of Animal Experimentation (CONCEA, 2013), as well as the CONCEA Guidelines for Euthanasia Practice (2013).

In addition, the study was designed and reported in compliance with the ARRIVE guidelines [22] and the PREPARE guidelines [23], ensuring methodological rigor, transparency, and reproducibility in the use of animal models. Experimental planning and data analysis adhered to the principles of the 3Rs—Reduction, Refinement, and Replacement—as defined by the National Centre for the Replacement, Refinement and Reduction of Animals in Research (NC3Rs) [24].

### 2.2. Animal Model Selection

The selection of an appropriate animal model should be guided by its biological relevance and its capacity to accurately reproduce specific aspects of the biological process under investigation. Accordingly, the model must exhibit sufficient validity, reliability, and reproducibility to fulfill the experimental objectives, while also considering the organism’s position within the phylogenetic hierarchy. When robust and translatable data can be obtained using small laboratory animals, such as rodents, their use is generally preferred due to ethical considerations, practical feasibility, and cost-effectiveness.

In the context of gingival mucosal wound healing, rodent models offer several advantages, including low maintenance and operational costs, ease of handling, and the ability to maintain strict control over experimental variables. Moreover, the use of genetically homogeneous strains contributes to the standardization of experimental outcomes and minimizes inter-individual biological variability. These characteristics render rodent models particularly well suited for histological, immunohistochemical, and biomolecular investigations of tissue repair and inflammatory responses [25,26,27].

### 2.3. Sample Size Calculation

Sample size estimation was performed using the Sealed Envelope software, based on an a priori statistical power analysis. Effect size estimates were derived from previously published studies evaluating gingival mucosal wound healing using the same animal model. The analysis determined that a minimum of five animals per experimental group was required to achieve a statistical power of 90% (β = 0.10) for the detection of statistically significant differences, assuming a two-sided significance level of 5% (α = 0.05).

### 2.4. Experimental Groups

In this study, the animals were divided into three groups and experimental time points as shown in Table 1.

### 2.5. Animal Characterization

In this study, 45 Wistar female rats (Rattus norvegicus albinus), weighing between 250 and 300 g, were used. The animals were obtained from the Laboratory Animal Facility (NAL) of the Federal Fluminense University (UFF), Niterói, Rio de Janeiro, Brazil. Throughout the experimental period, the animals were housed in mini isolators with autoclaved pine shavings used as bedding, at a maximum density of five animals per unit, in order to ensure appropriate hygienic conditions and animal welfare.

The animals received a standard laboratory diet (Nuvilab^®^, Belo Horizonte, MG, Brazil) in crushed form, provided ad libitum and replaced daily to prevent microbial contamination. Water was supplied ad libitum via glass bottles fitted with stainless steel drinking spouts.

Environmental conditions were controlled. The ambient temperature of the vivarium was maintained between 16 °C and 20 °C to ensure environmental stability throughout the experimental period. Although this range is slightly below the thermoneutral zone described for rodents, it remains within acceptable limits for adult Wistar rats maintained under standard laboratory housing conditions. Rats possess effective physiological and behavioral thermoregulatory mechanisms that allow them to maintain metabolic homeostasis across a relatively broad range of ambient temperatures when provided with appropriate bedding, shelter, and group housing. Previous guidelines and experimental studies indicate that adult laboratory rats can be safely housed within this temperature range without compromising physiological stability or experimental outcomes [28]. Therefore, the environmental conditions used in the present study were considered adequate to maintain animal welfare and metabolic equilibrium during the experimental procedures.

A controlled photoperiod of 12 h light and 12 h dark was implemented.

All housing and husbandry conditions were designed to ensure thermal, sanitary, and behavioral comfort, thereby preserving animal health and well-being. Experimental procedures and animal handling were conducted in strict accordance with the ethical principles of the 3Rs—Reduction, Refinement, and Replacement—as established by the NC3Rs program, with measures adopted to minimize the number of animals used as well as pain, stress, and suffering throughout the study [24].

### 2.6. Anesthesia and Surgical Procedures

All experimental procedures were performed under general anesthesia, following established protocols to ensure adequate analgesia and to minimize animal discomfort. Prior to surgery, the animals were subjected to a six-hour solid food fast, with free access to water. Body weight was recorded immediately before anesthesia induction using a precision digital scale (Gehaka^®^ BG 4001, Sao Paulo, SP, Brazil) to allow calculation of individualized anesthetic dosages based on body mass. General anesthesia was induced via intraperitoneal administration of ketamine hydrochloride (Francotar^®^; Virbac, Brazil) at a dose of 100 mg/kg in combination with xylazine hydrochloride (Sedazine^®^; Fort Dodge, IA, USA) at a dose of 10 mg/kg. The surgical procedure was initiated only after confirmation of a deep anesthetic plane, as evidenced by the complete absence of nociceptive reflexes.

**Figure 1:**
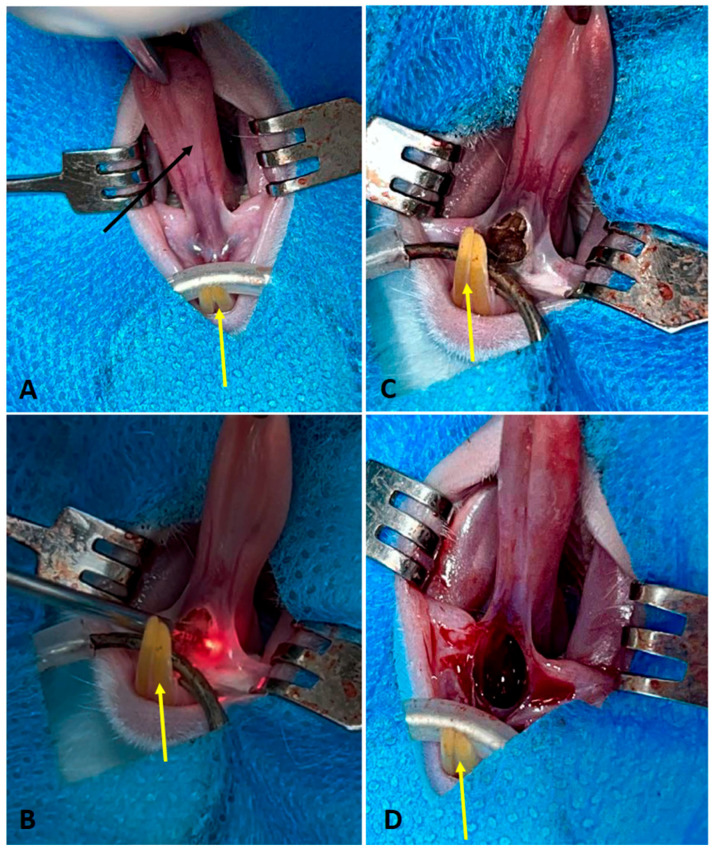
(**A**) Clinical aspect of the lingual frenulum of a Wistar rat. (**B**) Surgery performed with high-power laser (Group 1). (**C**) Surgery performed with electric scalpel (Group 2). and (**D**) Surgery performed with conventional scalpel (Group 3). Black arrow: lingual frenulum. Yellow arrows: lower incisors.

Following anesthesia induction, antisepsis of the oral cavity was performed using an aqueous solution of 0.5% chlorhexidine digluconate. The animals were then positioned in dorsal recumbency on the operating table (Figure 1), and sterile surgical fields were established. Lingual frenectomy was performed according to the protocol specific to each experimental group (Table 2). Briefly, hemostatic forceps were applied to the apex of the tongue to provide traction and adequate exposure of the operative field, and the lingual frenulum was incised at its thinnest connective tissue portion between the inferior alveolar process and the ventral surface of the tongue. After completion of the procedure, the animals were continuously monitored during the immediate postoperative period for up to two hours. Postoperative analgesia was achieved by subcutaneous administration of tramadol hydrochloride at a dose of 15 mg/kg, repeated at 12-h intervals in accordance with the established analgesic protocol.

### 2.7. Euthanasia of Animals

At the conclusion of the predetermined experimental periods, the animals were euthanized by intraperitoneal administration of an overdose of sodium thiopental at a dose of 150 mg/kg body weight. To minimize potential discomfort associated with administration of the euthanasia agent, 2% lidocaine was administered subcutaneously at a dose of 2 mg/kg prior to thiopental injection. Death was confirmed by the absence of respiratory movements, cardiac activity, and corneal reflexes. Following confirmation of death, the carcasses were placed in appropriately labeled biological waste containers and stored in a designated freezer at the Animal Experimentation Laboratory under controlled conditions until collection. Final disposal was performed by a licensed third-party company contracted by the university, in accordance with institutional and regulatory guidelines for the incineration of biological waste.

### 2.8. Histological Processing of Obtained Samples

The samples were initially fixed in 3.7% buffered formalin solution (phosphate buffer, pH 7.4) for a period of 48 h. Subsequently, they underwent standard histological processing at the Applied Biotechnology Laboratory of the School of Dentistry at the Federal Fluminense University. After fixation, the samples were gradually dehydrated in ethanol solutions of increasing concentrations (70%, 80%, 90%, and 100%), remaining for 1 h in each concentration. Clearing was performed with two successive baths of xylene, each lasting 1 h. The embedding step was conducted by sequential immersion in two paraffin baths (I and II), also lasting 1 h each, followed by embedding in paraffin with a melting point between 58 °C and 60 °C, using preheated metal molds. From the resulting blocks, longitudinal histological sections of 5 µm thickness were cut using a precision microtome. The sections were mounted on glass slides and stained with Hematoxylin and Eosin (H&E) for subsequent microscopic analysis.

### 2.9. Descriptive Microscopic Analysis

The stained histological sections were examined using a binocular bright-field light microscope (Olympus BX43; Olympus Corporation, Tokyo, Japan), equipped with an 80A filter (for color temperature correction) and plan-apochromatic objectives (4×, 10×, 20×, and 40×, N.A. 0.65). Selected images were captured by a digital camera through the cellSens software V 4.4.1 (Olympus Corporation, Tokyo, Japan). Histological descriptions and analyses were conducted to observe and quantify inflammatory cells (neutrophils, plasma cells, lymphocytes, macrophages, and multinucleated giant cells), tissue necrosis, fibrosis, fatty infiltration, and neovascularization, according to ISO Standard 10993-6:2016 [29]. Microscopic analysis was performed at the Associated Laboratory for Clinical Research in Dentistry (LPCO) of FOUFF.

### 2.10. Statistical Analysis

The statistical analysis aimed to evaluate the influence of the type of instrument used for the surgical incision (cold scalpel, electric scalpel, or laser) and the postoperative time (1, 3, or 7 days) on the tissue inflammatory response, according to the histological criteria established by ISO 10993-6:2016. The experimental design followed a full factorial arrangement (3 × 3) with two fixed independent factors: treatment type and postoperative time. Animals were randomly assigned to only one specific combination of technique and time, without overlap between groups. Each experimental cell (e.g., electric scalpel at day 3) consisted of five independent animals (n = 5 per group), totaling 45 subjects. For each animal, five histological slides representing different fields of the incision area were analyzed, and the final inflammatory score was obtained as the intra-animal average of these five fields. Based on this design, analyses were performed on the mean score per animal, resulting in a single value per subject. Due to the ordinal nature of the semi-quantitative reactivity scores assigned according to the histological parameters defined in ISO 10993-6:2016, the non-parametric Kruskal–Wallis test was used. This test was conducted separately to evaluate the effect of time (1, 3, and 7 days) within each treatment group (cold scalpel, electric scalpel, and laser) and the effect of the instrument type at each experimental time. When the test indicated a statistically significant difference, Dunn’s multiple comparisons post hoc test with correction for multiple comparisons was applied to identify the pairs responsible for the difference. The significance level adopted was α = 0.05.

## 3. Results

### 3.1. Descriptive Histological Analysis

Histological evaluations were performed according to the experimental groups and time points in interest to assess the tissue response according to the type of surgical procedure performed.

#### 3.1.1. Experimental Period: 1 Day

**Figure 2:**
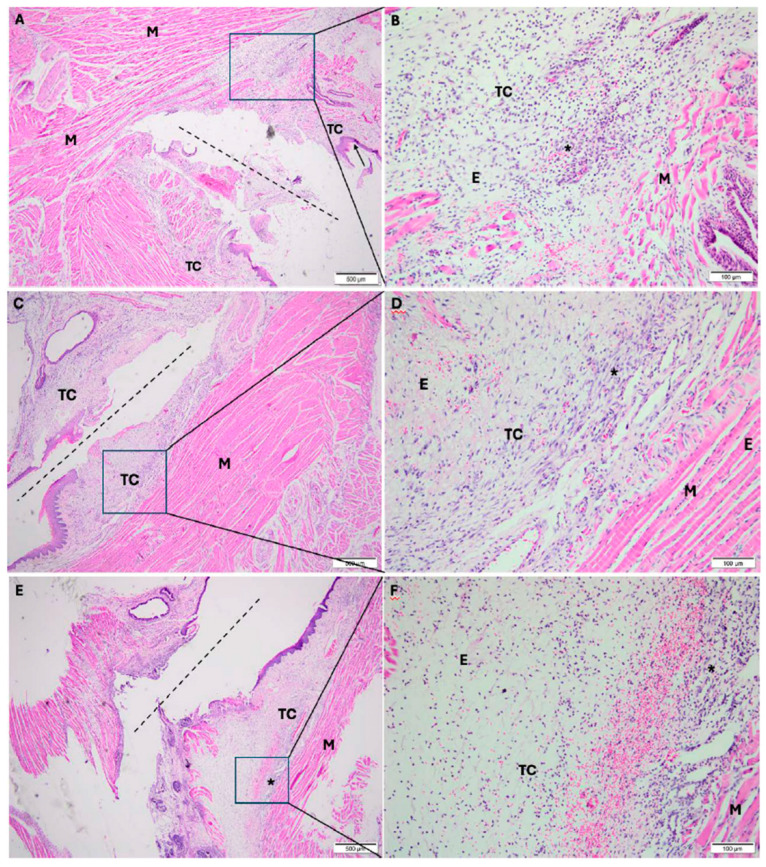
Photomicrographs taken 1 day after surgery. (**A**,**B**): frenectomy performed with a cold scalpel; (**C**,**D**): frenectomy performed with an electrosurgical scalpel; and (**E**,**F**): frenectomy performed with a diode laser. (**A**,**C**,**E**): 40× magnification; (**B**,**D**,**F**): 200× magnification. TC: connective tissue; M: muscle; E: edema; * inflammatory infiltrate. Dash line: incision. Staining: Hematoxylin and Eosin.

In the cold scalpel group, the frenectomy area (dashed line) showed oral mucosa composed of connective tissue, partially covered by stratified squamous epithelium, with foci of intense, diffuse, mixed inflammatory infiltrate (acute and chronic), and interstitial edema permeating collagen and muscle fibers (Figure 2A,B).

In the electrosurgical scalpel group, the frenectomy area showed oral mucosa composed of connective tissue, with foci of intense mixed inflammatory infiltrate (acute and chronic) and interstitial edema permeating collagen and muscle fibers (Figure 2C,D).

In the diode laser group, the frenectomy area showed oral mucosa composed of connective tissue, partially covered by stratified squamous epithelium, exhibiting foci of intense mixed inflammatory infiltrate (acute and chronic), interstitial edema permeating collagen and muscle fibers (Figure 2E,F).

#### 3.1.2. Experimental Period: 3 Days

**Figure 3:**
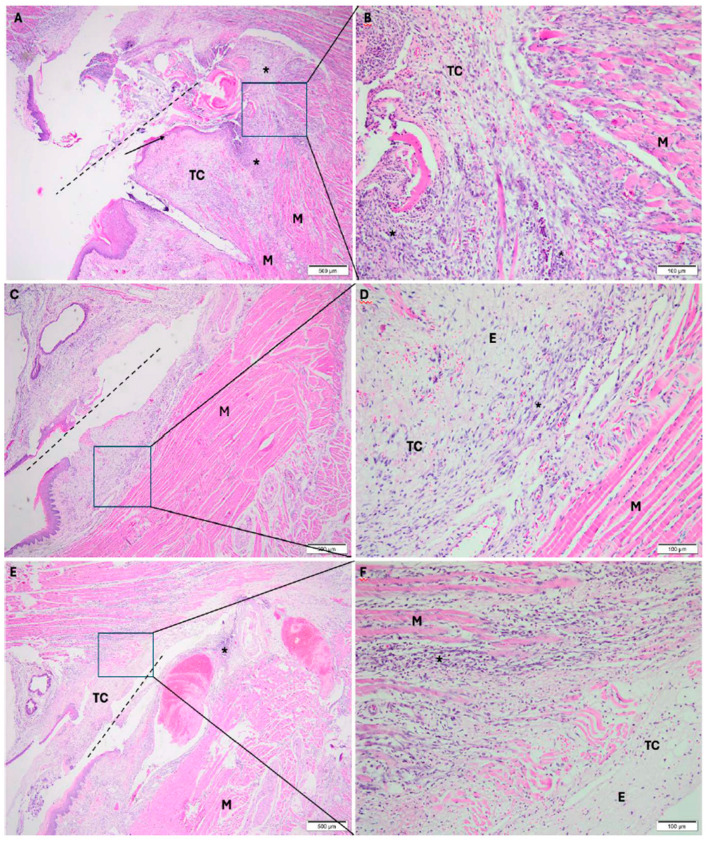
Photomicrographs taken 3 days after surgery. (**A**,**B**): frenectomy performed with a cold scalpel; (**C**,**D**): frenectomy performed with an electrosurgical scalpel; and (**E**,**F**): frenectomy performed with a diode laser. (**A**,**C**,**E**): 40× magnification; (**B**,**D**,**F**): 200× magnification. TC: connective tissue; M: muscle; E: edema; * inflammatory infiltrate. Dash line: incision. Black arrow: stratified squamous epithelium. Staining: Hematoxylin and Eosin.

In the cold scalpel group, the incision area showed oral mucosa composed of connective tissue, partially covered by stratified squamous epithelium, exhibiting foci of intense mixed inflammatory infiltrate (acute and chronic) permeating collagen and muscle fibers (Figure 3A,B).

In the electrosurgical scalpel group, the frenectomy area showed oral mucosa composed of connective tissue, partially covered by stratified squamous epithelium, with foci of moderate inflammatory infiltrate, predominantly chronic; in deeper layers, muscle fibers with scant edema were observed (Figure 3C,D).

In the diode laser group, the frenectomy area showed oral mucosa composed of connective tissue, partially covered by stratified squamous epithelium, with foci of moderate chronic inflammatory infiltrate; in deeper layers, muscle fibers with scant edema were observed (Figure 3E,F).

#### 3.1.3. Experimental Period: 7 Days

**Figure 4:**
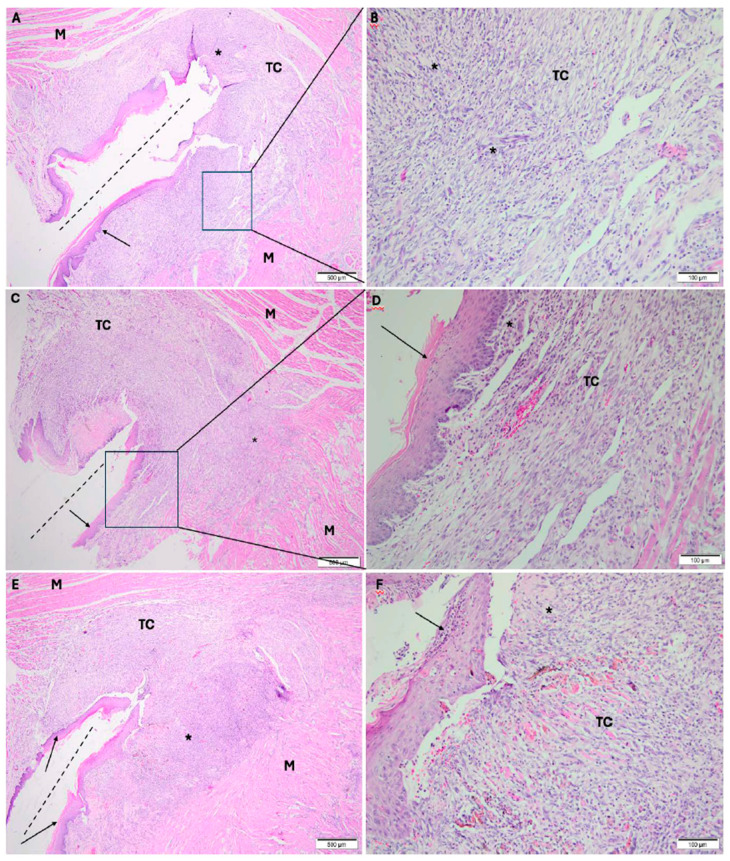
Photomicrographs taken 7 days after surgery. (**A**,**B**): frenectomy performed with a cold scalpel; (**C**,**D**): frenectomy performed with an electrosurgical scalpel; and (**E**,**F**): frenectomy performed with a diode laser. (**A**,**C**,**E**): 40× magnification; (**B**,**D**,**F**): 200× magnification. TC: connective tissue; M: muscle; E: edema; * inflammatory infiltrate; dash line: incision. black arrow: stratified squamous epithelium. Staining: Hematoxylin and Eosin.

In the cold scalpel group, the frenectomy area showed oral mucosa composed of fibrocellular connective tissue, almost entirely covered by stratified squamous epithelium, exhibiting diffuse inflammatory infiltrate predominantly chronic; in deeper layers, muscle fibers with scant edema were observed (Figure 4A,B).

In the electrosurgical scalpel group, the frenectomy area showed oral mucosa composed of fibrocellular connective tissue, almost entirely covered by stratified squamous epithelium, with a diffuse, predominantly chronic inflammatory infiltrate; in deeper layers, muscle fibers with scant edema were observed (Figure 4C,D).

In the diode laser group, the frenectomy area showed oral mucosa composed of fibrocellular connective tissue, covered by stratified squamous epithelium, with a diffuse predominantly chronic inflammatory infiltrate; in deeper layers, muscle fibers were observed (Figure 4E,F).

### 3.2. Statistical Analysis Results

According to ISO 10993-6:2016, the reactivity score was calculated based on the weighted sum of the scores assigned to the different types of inflammatory cells, considering the respective weighting factors defined by the standard. This procedure allows histological observations to be converted into a single index of tissue reaction intensity, ensuring comparability among experimental groups. For the individual analysis, each cell type (polymorphonuclear cells, lymphocytes, plasma cells, macrophages, and giant cells) received a semiquantitative score according to the criteria established in the standard, reflecting the relative intensity of the inflammatory infiltrate.

The Kruskal–Wallis test indicated that, for the cold scalpel and the electrosurgical scalpel, there was no significant difference among the experimental periods (*p* = 0.1230 and *p* = 0.2459, respectively). In contrast, for the diode laser group, a significant difference among periods was observed (*p* = 0.0020), and Dunn’s post hoc test revealed that the score on day 7 was significantly higher than on days 1 and 3 (*p* = 0.0397 and *p* = 0.0139, respectively). In the time-based analysis, no difference among instruments was observed on day 1 (*p* = 0.3901). On day 3, a significant difference was detected (*p* = 0.0381), but not pairwise as the comparison reached significance after adjustment. On day 7, there was a highly significant difference among instruments (*p* = 0.0003), with the laser showing higher scores compared with the electrosurgical scalpel (*p* = 0.0054).

**Figure 5:**
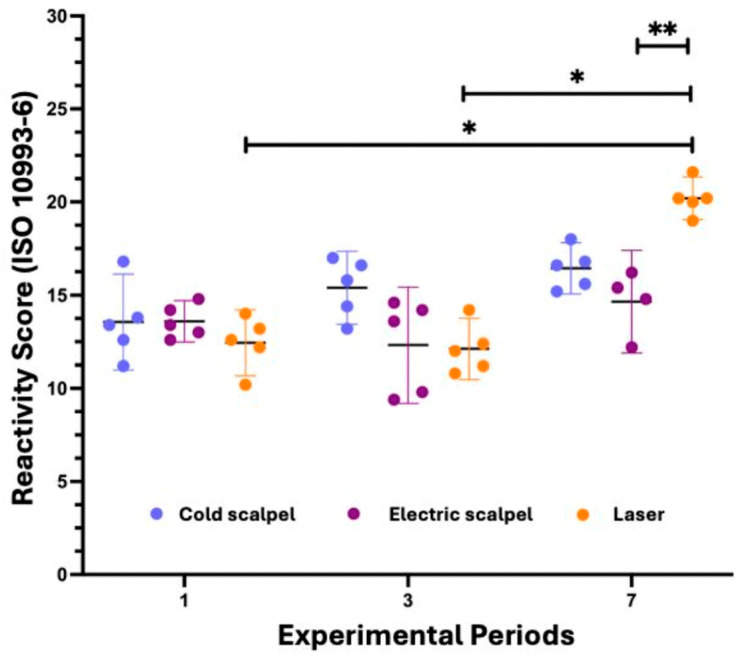
Reactivity scores (ISO 10993-6) at the different experimental periods (1, 3, and 7 days) for each evaluated instrument: cold scalpel (light blue), electrosurgical scalpel (red), and laser (orange) Horizontal bars represent the mean of the median scores with a 95% confidence interval. Statistical differences were assessed using the Kruskal–Wallis test followed by Dunn’s post hoc test (* *p* < 0.05; ** *p* < 0.01). A significant increase in the score was observed in the laser group on day 7 compared with the other periods, as well as a significant difference between the laser and the electrosurgical scalpel on day 7.

Overall, these results indicate that the inflammatory response was similar among treatments at the early time points; however, the laser exhibited a higher reactivity score on day 7, significantly differing from the electrosurgical scalpel (Figure 5).

Statistical analysis demonstrated significant time-related effects for all evaluated groups regarding polymorphonuclear (PMN) cell infiltration. In the cold scalpel group, a significant reduction in scores was observed between days 1 and 7 (*p* = 0.0362). Similar results were found in the electrosurgical scalpel group, which also showed a decrease in scores between days 1 and 7 (*p* = 0.0298). Likewise, the laser group exhibited a significant reduction in PMN counts, with lower scores at day 7 compared with day 1 (*p* = 0.0217). When comparing the different instruments at each experimental period, no significant differences were detected at day 1 (*p* = 0.4505) or day 7 (*p* = 0.1669). However, at day 3, a significant difference was observed between the cold scalpel and electrosurgical scalpel groups (*p* = 0.0412), with greater PMN infiltration in the cold scalpel group. Overall, these findings indicate that the acute inflammatory response mediated by PMNs was more intense at the early stages of the healing process, with a progressive reduction up to day 7 in all experimental groups, although with slight variations in the temporal pattern among the different instruments.

Analysis of lymphocytic infiltration revealed no significant differences associated with either time or the type of surgical instrument. Within each group (cold scalpel, electrosurgical scalpel, and laser), lymphocyte scores remained stable between days 1, 3, and 7, with no statistically relevant variations (cold scalpel: *p* = 0.3297; electrosurgical scalpel: *p* = 0.3007; laser: *p* = 0.4505). Similarly, when comparing the three instruments at each experimental period, no significant differences were observed among the groups (day 1: *p* = 0.8002; day 3: *p* > 0.9999; day 7: *p* > 0.9999). Overall, the data indicate that the chronic inflammatory response mediated by lymphocytes was mild and homogeneous, with no significant influence of the evaluation time or the incision method employed.

Statistical analysis of macrophage infiltration showed significant time-related differences only in the cold scalpel group (*p* = 0.0044). In this group, an increase in macrophage scores was observed between days 1 and 7 (*p* = 0.0058), indicating progressive cellular infiltration over the experimental period. In contrast, no statistically significant differences were identified among the evaluated time points in the electrosurgical scalpel (*p* = 0.0909) and laser (*p* = 0.0892) groups. When the three instruments were compared at each experimental period (1, 3, and 7 days), no significant differences were observed among the groups (*p* > 0.05 in all cases).

**Figure 6:**
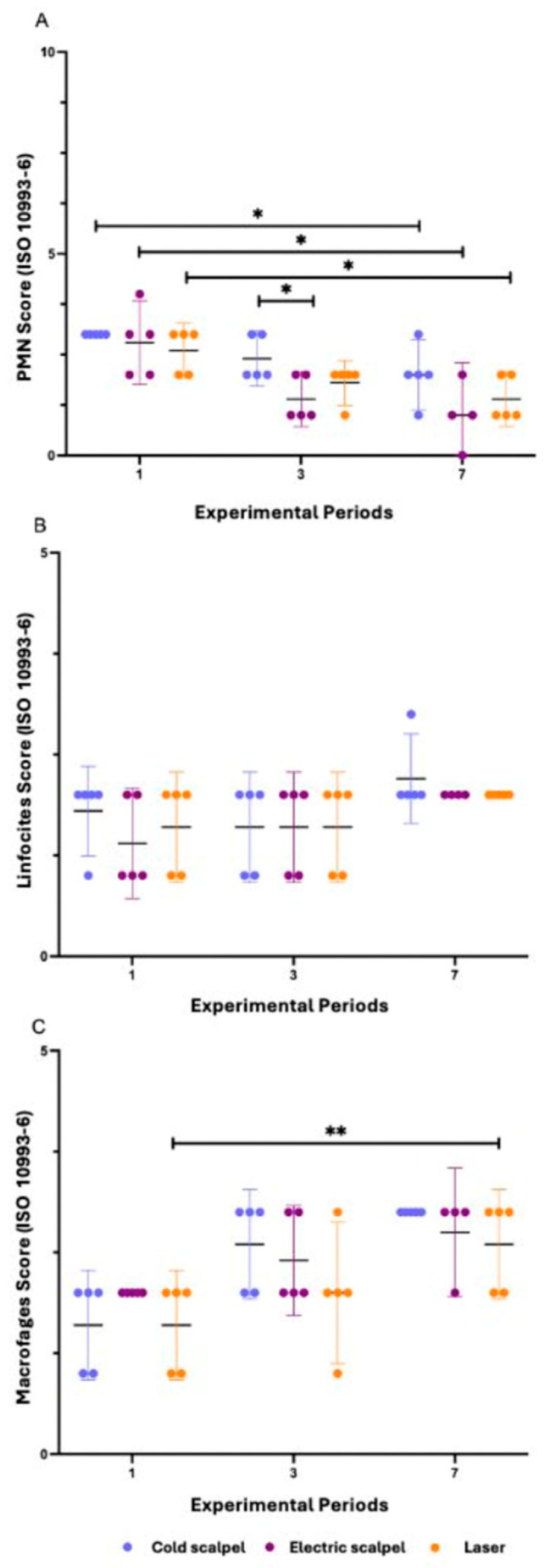
Inflammatory infiltrate in subcutaneous tissue evaluated according to ISO 10993-6 at different experimental periods (1, 3, and 7 days) after incision with a cold scalpel, electrosurgical scalpel, and laser. (**A**) Polymorphonuclear (PMN) score: a significant reduction was observed between days 1 and 7 in all groups, in addition to a significant difference between the cold scalpel and electrosurgical scalpel at day 3. (**B**) Lymphocyte score: no significant differences related to time or type of surgical instrument were detected. (**C**) Macrophage score: a significant increase between days 1 and 7 was identified only in the cold scalpel group. Data are presented as the mean of the median scores with a 95% confidence interval. Statistical differences were analyzed using the Kruskal–Wallis test followed by Dunn’s post hoc test. * *p* < 0.05, ** *p* < 0.01.

Additionally, plasma cells were rare, and giant cells were not identified in any of the analyzed groups. Overall, the results suggest that the macrophage response exhibited greater temporal variation only in the cold scalpel group, whereas the electrosurgical scalpel and laser groups showed more stable behavior across the different evaluation periods (Figure 6).

## 4. Discussion

Contemporary scientific literature in the medical and dental fields has provided consistent evidence regarding the clinical benefits associated with the use of high-power lasers in surgical procedures involving soft tissues. Among the main advantages reported are efficient hemostasis, greater surgical precision, reduced intraoperative pain and bleeding, as well as a decreased need for sutures and postoperative analgesic medications in the immediate postoperative period. However, factors such as the high cost of equipment and the requirement for specialized technical training to ensure safe and effective use of the technology remain relevant barriers to its widespread incorporation into routine clinical practice [30]. The growing demand for minimally invasive surgical techniques has driven the adoption of energy-based technologies, such as lasers, in both medicine and dentistry. In the Brazilian context, the implementation of the “Tongue-Tie Test” as a mandatory protocol in maternity hospitals since 2014 has resulted in a significant increase in the early diagnosis of ankyloglossia in neonates, directly impacting the frequency of indications for frenectomies in this population. This scenario, combined with technological advances and increased accessibility of laser devices, has favored their adoption as an alternative to the conventional cold scalpel technique.

It is important to acknowledge that surgical technique represents a critical variable that can influence clinical outcomes. While this study focused on tissue-level histological and histomorphometric analyses, differences between procedures such as frenotomy—entailing simple division of the mucosal frenulum—and more extensive approaches like frenuloplasty, which involves posterior dissection and mucosal closure, may impact both healing dynamics and functional outcomes. Frenuloplasty is often indicated for more severe or posterior ankyloglossia, whereas frenotomy may suffice for primarily mucosal frenula [1] Therefore, the choice of surgical technique should be considered when interpreting the translational relevance of these findings to clinical practice.

High-power lasers promote tissue cutting and coagulation through the conversion of light energy into heat, resulting in an increase in local temperature and tissue vaporization. The efficacy and safety of the procedure are directly related to the physicochemical characteristics of the equipment, particularly the wavelength and its interaction with different tissue components, which decisively influences the clinical and histopathological effects observed [31]. Despite the widely reported clinical benefits, the literature remains limited regarding detailed descriptions of the histological changes induced by these devices, especially in oral mucosal tissues. Studies suggest that thermal sources, such as electrosurgical scalpels and high-power lasers, may cause greater thermal damage and disruption of tissue architecture, potentially compromising tissue repair and favoring fibrosis formation or recurrence [32].

A large proportion of published studies focuses on the evaluation of clinical parameters, such as postoperative pain, intraoperative bleeding, and recurrence rates [33]. Aras et al. demonstrated that the Er:YAG laser can, in some cases, be used without local anesthesia, unlike the diode laser, which still requires anesthesia, especially in lingual frenectomies. In the present study, however, all animals were subjected to general anesthesia to ensure animal welfare and standardization of the experimental protocol. Therefore, pain assessment was not included among the analyzed outcomes.

Most studies found in the literature on laser-assisted frenectomies emphasize clinical aspects, such as patients’ subjective perception of postoperative pain, control of intraoperative bleeding, and recurrence rates [33]. Regarding the histological aspects of healing, Mazzoni et al. [13], in a randomized clinical trial, reported a higher incidence of scar fibrosis in neonates undergoing diode laser frenectomy when compared with electrosurgical scalpel procedures. This finding reinforces the relevance of studies that investigate, in a controlled manner, the histological effects of laser devices, particularly in vulnerable populations such as newborns. In the present study, the fibrotic phase of repair was not evaluated, as the animals were euthanized at 1, 3, and 7 postoperative days, with the focus placed on the early phases of the inflammatory response and the tissue regeneration process. Mazzoni et al. [13] also emphasize the clinical efficacy and comfort provided by laser use in pediatric procedures, although they point out the lack of consensus regarding the ideal power settings for application in neonates. The protocol adopted in the present study was based on parameters previously used in experimental rodent models [33].

Similarly, to the findings of the present study, Oliveira et al. [34] observed, in a comparative study between diode laser and electrosurgical scalpel on the tongues of rats, that both groups exhibited healing by secondary intention, supporting the absence of significant differences in the overall pattern of tissue repair. Tenore et al in a systematic review, concluded that the application of lasers, such as CO_2_ and diode lasers, does not preclude histopathological analysis of tissue samples [35]. The findings of the present study corroborate this conclusion, as morphological analysis remained feasible after laser application, with sufficient preservation of anatomical and histological structures for evaluation.

Murias et al. [29] also demonstrated clinical advantages of laser use in frenectomies, such as shorter surgical time, reduced need for anesthesia and suturing, and a lower incidence of postoperative complications. In the present experiment, faster execution of the procedure and the absence of suturing were observed in the laser-treated group. However, the analysis of variables such as the need for anesthesia or analgesic medication was not feasible due to the standardized use of general anesthesia in all groups and the uniform postoperative pharmacological protocol. In contrast, Bilder et al. [36] reported that, despite the technical advantages of laser use, patients who underwent diode laser procedures experienced greater postoperative pain and increased tissue damage compared with those treated with electrosurgical scalpel. These findings, however, were not confirmed in the present study, in which no statistically significant differences were observed among groups regarding body weight loss, inflammatory infiltration, presence of necrosis, fibrosis, or adipose infiltrate during the evaluated periods (1, 3, and 7 days).

Verma et al. [37] compared frenectomy techniques performed with conventional scalpel, laser, and electrosurgery, clinically assessing postoperative pain and discomfort. Their results showed that on the first postoperative day, the laser-treated group experienced less pain and discomfort during mastication, whereas on the seventh day, the electrosurgery group showed the most favorable outcomes in this regard. Although the present study adopted the same evaluation periods, discomfort was assessed indirectly through observation of animal behavior and changes in body weight, since this was an animal model.

A clinical investigation compared the use of blue diode laser with two conventional surgical approaches—the infrared diode laser and the quantic molecular resonance (QMR) scalpel—for the excision of benign oral lesions. The authors reported that all three techniques enabled adequate tissue sampling for histopathological diagnosis, demonstrating their suitability for surgical management of these lesions. However, the blue diode laser showed advantages, including effective hemostasis and a reduced risk of intraoperative bleeding, while maintaining limited thermal damage to surrounding tissues. These findings suggest that blue diode laser technology may represent a promising alternative to traditional surgical modalities for the treatment of benign oral lesions.

In the present study, the results demonstrated that the tissue samples obtained were adequate for histological evaluation, consistent with the findings reported in the previously cited study. No differences were observed among the surgical techniques regarding the quality of the specimens. These findings indicate that lingual frenulum biopsies obtained with the different surgical modalities were suitable for both histological and histomorphometric analyses, allowing reliable assessment of the tissue response. Histological and histomorphometric analyses did not reveal statistically significant differences among groups regarding immediate tissue repair. However, a slight increase in polymorphonuclear (PMN) cell counts was observed in the cold scalpel group on the first postoperative day, suggesting a more pronounced acute inflammatory response. In a clinical scenario, this response could be associated with greater postoperative pain or discomfort. Between the electrosurgical scalpel and laser groups, the initial inflammatory response was similar. At the end of seven days, all three groups exhibited a similar healing pattern, with a slight increase in the inflammatory score in the laser-treated group.

These findings partially contrast with expectations generated by clinical evidence attributing advantages to laser use, such as reduced surgical trauma and accelerated healing. The similarity in the initial inflammatory response among the techniques suggests that the thermal effect of the laser may induce histological changes which, depending on the intensity and duration of exposure, have the potential to negatively impact the repair process, predisposing the patient to fibrosis formation and possible recurrences, as described in previous studies.

The findings of this study should be interpreted considering several limitations that restrict direct clinical extrapolation. First, the use of an animal model, although valuable for controlled histological evaluation, does not fully replicate the anatomical complexity, healing dynamics, and functional biomechanics of human oral tissues. Second, the observation period focused on early healing events, which limits the ability to assess later stages of tissue remodeling, including fibrosis and scar maturation. Because scar formation and collagen reorganization occur during later phases of wound healing, the present design cannot determine the long-term fibrotic outcomes of the procedures. Additionally, parameters directly associated with thermal effects, such as the depth of thermal necrosis and quantitative collagen deposition, were not measured. These variables could provide further insight into the biological impact of diode laser–tissue interaction and its potential influence on long-term tissue remodeling. Therefore, while the present results contribute important information regarding early inflammatory and histological responses, caution is warranted when translating these findings to clinical practice, and further studies with longer follow-up and expanded histological analyses are necessary to better characterize long-term outcomes.

The detailed histological evaluation in this study underscores the critical role of polymorphonuclear cells, particularly neutrophils, in early tissue repair. Neutrophils are essential not only for initiating the inflammatory response but also for regulating the balance between effective tissue repair and fibrosis, facilitating necrotic tissue clearance, and supporting the transition to the proliferative phase [38,39]. In this context, the higher number of neutrophils observed in cold scalpel incisions during the early stages of healing may have important translational implications. Enhanced early neutrophil recruitment likely promotes organized tissue repair and timely resolution of inflammation, which could explain why cold dissection is often associated with a lower incidence of excessive fibrosis and unexpected scar formation in clinical practice. Conversely, laser-induced thermal effects may reduce or delay neutrophil infiltration, prolong the inflammatory phase, and influence tissue remodeling, highlighting the interplay between the incision modality, early cellular dynamics, and long-term healing outcomes.

It is important to distinguish between the favorable clinical perception of diode laser surgery and the underlying biological tissue response. Clinically, diode lasers are often associated with advantages such as improved intraoperative hemostasis, reduced need for sutures, and enhanced surgical visibility, which may lead to the perception of superior healing. However, these practical benefits do not necessarily correspond to a reduced cellular inflammatory response during the healing process. In the present study, histological analysis revealed a higher reactivity score in the laser group at day 7, suggesting a prolonged inflammatory phase compared with conventional scalpel incisions. A possible explanation lies in the mechanism of laser–tissue interaction. Diode lasers generate thermal energy that induces coagulation, protein denaturation, and a superficial zone of thermal necrosis at the incision margins. The presence of this devitalized tissue may delay its clearance by inflammatory cells and macrophages, thereby prolonging the inflammatory phase and influencing early repair dynamics. In contrast, cold scalpel incisions produce sharply defined tissue margins with minimal collateral damage, allowing more rapid progression from inflammation to the proliferative phase of healing. This difference in tissue injury patterns may explain why the clinical advantages of laser surgery do not always correspond to a reduced histological inflammatory response during early wound healing.

These findings highlight the importance of further in vivo studies aimed at establishing evidence-based surgical protocols, with parameters adjusted to the specific characteristics of each age group and clinical condition. In addition, the relevance of the practitioner’s learning curve is emphasized as a critical factor for the efficacy and safety of laser use in surgical procedures.

## 5. Conclusions

Based on the histological and histomorphometric findings related to the initial tissue healing process following lingual frenectomy in rats, it can be concluded that no statistically significant differences were observed in the inflammatory response among the groups and postoperative periods.

However, further investigation is still required to deepen the understanding of the biological effects induced by different laser emission parameters on oral tissues. Additional studies are essential for the standardization of evidence-based clinical protocols, aiming to ensure greater safety, efficacy, and predictability of outcomes.

## Figures and Tables

**Table 1:** Distribution of the number of animals according to groups and experimental periods.

Groups	Surgical Techniques	Animals	Experimental Periods
1	Laser	15	1, 3 and 7 days
2	Electric scalpel	15	1, 3 and 7 days
3	Cold scalpel	15	1, 3 and 7 days

**Table 2:** Protocol and equipment used for each surgical technique according to the experimental groups.

Groups	Surgical Techniques	Protocol
1	Laser	Diode laser (GaAlAs, wavelength 980 ± 20 nm, power 1 W–Thera Lase Surgery, DMC, São Paulo, Brazil) delivered through a flexible 400 µm optical fiber in continuous mode, in contact with the oral mucosa. *
2	Electric scalpel	Electrosurgical unit with a needle-tip electrode (Medcir BO 560 model), in cutting mode at intensity level 3.
3	Cold scalpel	No. 3 scalpel handle with a 15C Bard-Parker blade.

* The diode laser (980 ± 20 nm, 1 W) delivered through a 400 µm optical fiber produced an estimated irradiance of approximately 794 W/cm^2^, corresponding to a fluence of 794 J/cm^2^ per second of irradiation at the fiber tip.

## Data Availability

The original contributions presented in this study are included in the article. Further inquiries can be directed to the corresponding authors.
